# Multimodal Personal Verification Using Likelihood Ratio for the Match Score Fusion

**DOI:** 10.1155/2017/9345969

**Published:** 2017-10-31

**Authors:** Long Binh Tran, Thai Hoang Le

**Affiliations:** ^1^Computer Science Department, University of Lac Hong, Dong Nai 810000, Vietnam; ^2^Computer Science Department, VNUHCM-University of Science, Ho Chi Minh City 700000, Vietnam

## Abstract

In this paper, the authors present a novel personal verification system based on the likelihood ratio test for fusion of match scores from multiple biometric matchers (face, fingerprint, hand shape, and palm print). In the proposed system, multimodal features are extracted by Zernike Moment (ZM). After matching, the match scores from multiple biometric matchers are fused based on the likelihood ratio test. A finite Gaussian mixture model (GMM) is used for estimating the genuine and impostor densities of match scores for personal verification. Our approach is also compared to some different famous approaches such as the support vector machine and the sum rule with min-max. The experimental results have confirmed that the proposed system can achieve excellent identification performance for its higher level in accuracy than different famous approaches and thus can be utilized for more application related to person verification.

## 1. Introduction

It is proven in the literature that personal verification systems using biometric modalities acquire outweighing advantages in terms of security and conveniences. Thus, there are now many biometric systems which are used widely, like face, facial thermograms, fingerprint, hand geometry, hand vein, iris, retinal pattern, signature, voice-print, and so on [1].

Currently, unibiometric systems, the systems working on single biometric traits, are rather popular in use. Despite their significant development, these systems still have some disadvantages that can curb their effectiveness in performance in terms of noise, limited level of freedom, intraclass variability, spoofing attack, unacceptable error rates, and so on. Some of these drawbacks, however, can be handled by systems using multiple biometrics including different sensors, multiple samples of the same biometrics, different feature representations, multiple algorithms, or multimodalities [2–4]. Among these, multimodal systems utilize multiple traits, physiological or behavioural, for enrollment and identification.

Multimodal biometric systems have been accepted by many professionals thanks to (1) their superior performance and (2) to overcome other limitations of unibiometric systems [3]. This leads to the hypothesis that our employment of multiple modalities (face, fingerprint, palm print, and hand shape) can conquer the limitations of the single modality-based techniques. Multimodal biometrics have many fusion levels [3], such as sensor level, feature level, matching score level, and decision level. With its efficiency and simplicity, fusion at score level becomes a preferable fusion technique [3, 5] although combining scores of different matchers with dissimilar nature and scale is a real challenge because the scores of different matchers can be either distance or dissimilarity measure. Finally, the match scores may follow different probability distributions, may provide quite different accuracies, and may be correlated. Techniques of fusing at score level are put in three groups: transformation-based score fusion [6–8], classifier-based score fusion [9, 10], and density-based score fusion [11, 12]. The last group is based on the likelihood ratio test and it requires explicit estimation of genuine and impostor match score densities. This scores density approach is based on the Neyman-Pearson theorem [13], which has the advantage that it directly achieves optimal performance at any desired operating point, provided the score densities are estimated accurately.

Our work aims at exploring effective ways to combine extracted multiple biometric features into templates for personal verification. To achieve this aim, we suggest an approach using Zernike Moment (ZM) and score level fusion technique based on likelihood ratio test and the finite Gaussian mixture model (LR –GMM) [14]. In this approach, ZM [15] is used to extract features of multimodal images (face, fingerprint, palm print, and hand shape). In this way, the basis function of ZM is defined on a unit circle and the center of the unit circle is set to coincide with the center of biometric images. This will extract more features, increasing the accuracy of personal verification. After matching, the performance of fusing the match scores using the likelihood ratio (LR) test and a finite Gaussian mixture model (GMM) for estimating the genuine and impostor score densities is examined. Finally, a decision is identified: an individual is genuine or impostor. Our proposed technique is also compared with the famous techniques such as support vector machine (SVM) and the sum rule with min-max and this comparison has shown outstanding results of the proposed technique.

The rest of this paper is about these contents: a depiction of the proposed system in Section 2; a description of the suggested methodology in Section 3; discussions about the experimental results in Section 4; and the paper conclusion in Section 5.

## 2. Proposed Multimodal System

**Figure 1:**
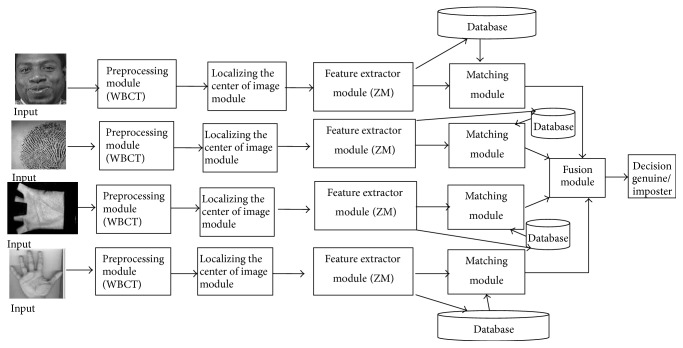
The chart of proposed personal verification system.

In our work, a system using multiple biometric traits (face, fingerprint, palm print, and hand shape images) for personal identification (Figure 1) is proposed consisting of two phases: enrollment and verification. Both phases include preprocessing biometric images with Wavelet-Based Contourlet Transform [16], localizing the center of image, extracting the feature vectors with ZM.

In the enrollment phase, the captured images are normalized and localizing the center of image for later feature extraction. Scores generated from the feature extractions are stored as templates in the database.

In the verification phase, the sets of feature scores obtained after image preprocessing, localizing the center of image and feature extraction, are supplied to the matching module where they are matched with the stored templates achieved in the enrollment phase, generating matching scores. These scores are fused and finally the chosen individual is identified.

Our proposed personal verification system is composed of five modules. In the first module, the image was preprocessed prior to the feature extraction. Our identification system used Wavelet-Based Contourlet Transform [16] to process the image normalization, noise elimination, illumination normalization, and so on. In the second module, Algorithms in [17–22] were used to locate the center of the best-fit ellipse in a face image, the reference point in a fingerprint image, the reference point in a palm print image, and the center of the elliptical model of a palm and each finger, and then the center of the unit circle of ZM is set to coincide with the reference point in a fingerprint image and with the center of the best-fit ellipse in a face image, the reference point in a palm print image, and the center of the elliptical model of a palm and each finger. In the third module, different features were extracted from the derived image normalization (feature domain) in parallel structure. To extract the features from the input images, Zernike Moment (ZM) was used. In the fourth module, the matching was carried out by Euclidean distance, based on the chosen features. The matching was done in each feature domain in parallel as Figure 1. In the last module, the outputs of each matcher were combined to construct the identification. In this paper, match score fusion method was selected for decision strategy and FVC2004 database [23], ORL database [24], PolyU database [25], and IIT Delhi database [26] were used for the experiment.

## 3. Methodology

In our paper, main modules of the proposed system including image preprocess, localizing the center of image, feature extraction, matching, and a multimodal biometric verification model are described in detail.

### 3.1. Image Preprocess

Due to the noise in biometric images, the quality of images may be poor and thus the identification cannot be done efficiently; therefore, this module aims at normalizing an image by reducing or eliminating some of its variations. To do it, Wavelet-Based Contourlet Transform (WBCT) [16] is used.

**Figure 2:**
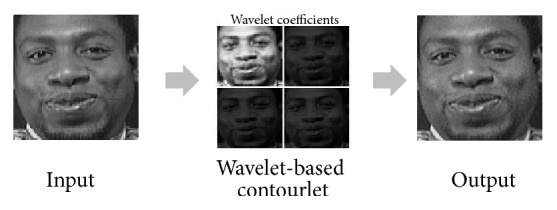
An example of the WBCT method.

Wavelet-Based Contourlet Transform in [16] is briefly described as follows: this system consists of two stages. In stage 1, an image is disintegrated into components of low frequency and high frequency, creating coefficients of various bands, which are later handled individually. Histogram equalization is applied to the approximation of the coefficients of low frequency. In stage 2, coefficients of high frequency are handled with a directional filter bank for smoothing the image edge. The image is normalized thanks to the coefficients modified by an inverse Wavelet-Based Contourlet Transform. The normalized image is enhanced in its contrast, its edges, and its details, all of which are necessary for further biometric image recognition (Figure 2).

See [16] for a detailed description.

### 3.2. Localizing the Center of Image

In this phase, we find the center of biometric images after normalization. This will extract more features and increase the accuracy of personal verification.

#### 3.2.1. The Reference Point of Fingerprint

**Figure 3:**
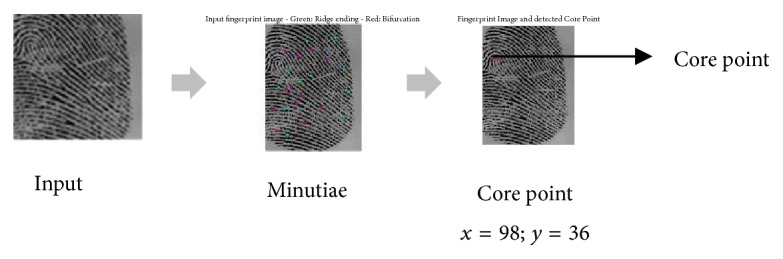
The reference point (the core point) on the fingerprint.

The reference point of a fingerprint is defined as the point of maximum curvature in the most internal crests. Usually, the core point is used as reference point. This point can be located by an algorithm which is briefly described as follows [17]:

(1) Choose a window with *w* × *w* size for the estimation of the orientation field *O*. A 7 × 7 mean filter is used in our work. The smoothed orientation field *O*′ at (*i*, *j*) is computed as follows:
  (1) $$\begin{array}{c} O^{\prime }\left(\;i,j\;\right)=\frac{1}{2}{tan}^{-1}\left(\;\frac{\Phi_{y}^{\prime }\left(i,j\right)}{\Phi_{x}^{\prime }\left(i,j\right)}\;\right). \end{array}$$
(2) Estimate *ε*, an image with the sine component of *O*′:
  (2) $$\begin{array}{c} \varepsilon \left(\;i,j\;\right)=\sin\left(\;O^{\prime }\left(\;i,j\;\right)\;\right). \end{array}$$
(3) Initialize *A*, a label image used for reference point indication.
(4) Identify the highest value in *A* and assign its coordinate to the core, that is, the reference point (Figure 3).

See [17] for a detailed description.

#### 3.2.2. The Center of Face Image

**Figure 4:**
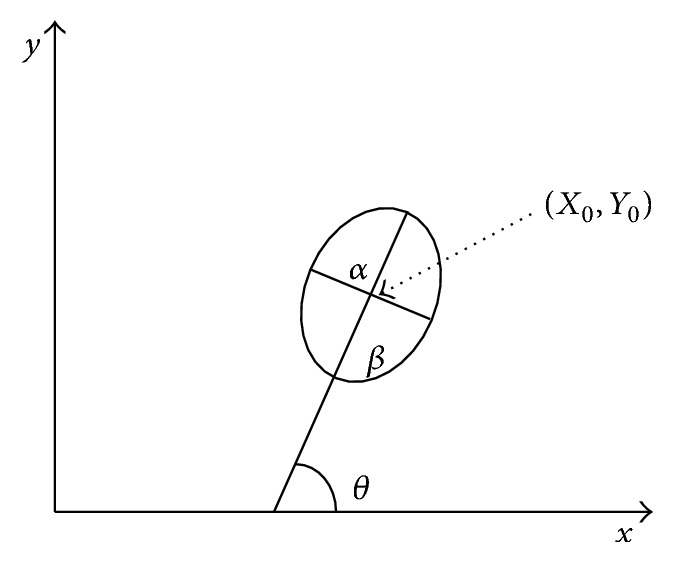
Face model based on ellipse model.

**Figure 5:**
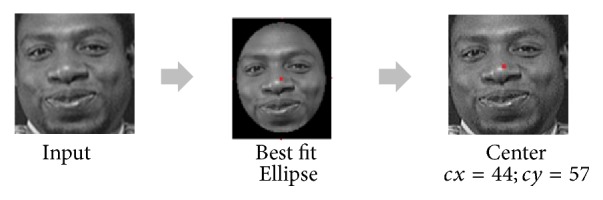
Localizing faces using best-fit ellipse.

In face image with frontal view, the face shape is approximate to an ellipse (Figure 5). In the algorithm, to find the best-fit ellipse [18], an ellipse model with five parameters is used; *X*_0_, *Y*_0_ denote the ellipse center;*θ* is the orientation; *α* and *β* are the minor and the major axes of the ellipse individually (Figure 4). Geometric moments are considered for the calculation of those five parameters.

The geometric moments of order *p*, *q* of a digital image are specified as

(3) $$\begin{array}{c} M_{pq}=\underset{x}{\sum }\underset{y}{\sum }f\left(\;x,y\;\right)x^{p}y^{q}, \end{array}$$

where *p*, *q* = 0,1, 2,… and *f*(*x*, *y*) denotes the gray scale value of the digital image at *x* and *y* locations. The origin is placed at the image center to capture the translation invariant central moments as summarized in the following equation:

(4) $$\begin{array}{c} \mu_{pq}=\underset{x}{\sum }\underset{y}{\sum }f\left(\;x-x_{0},y-y_{0}\;\right){\left(\;x-x_{0}\;\right)}^{p}{\left(\;y-y_{0}\;\right)}^{q}, \end{array}$$

where *x*_0_ = *M*_10_/*M*_00_ and *y*_0_ = *M*_01_/*M*_00_ represent the centers of the joined components of which center of gravity indicates the ellipse center. The orientation *θ* of the ellipse is estimated by the least moment of inertia [19, 20]

(5) $$\begin{array}{c} \theta =\frac{1}{2}\arctan\left(\;\frac{2\mu_{11}}{\mu_{20}-\mu_{02}}\;\right), \end{array}$$

where *μ*_*pq*_ is the central moment of joined components (4). By the least and the greatest moment of inertia of an ellipse is defined as

(6) IMin=∑x∑yx−x0cos⁡θ−y−y0sin⁡θ2,IMax=∑x∑yx−x0sin⁡θ−y−y0cos⁡θ2;

the lengths of the major and minor axes are computed as

(7) α=1πIMax3/IMin1/8,β=1πIMin3/IMax1/8.

See [19, 20] for a detailed description.

#### 3.2.3. The ROI of Palm Print

In this phase, we will find the region of interest (called ROI) in the palm table. The ROI is defined in square shape and it contains sufficient information to represent the palm print for further processing. The outline of the ROI could be obtained as follows [21].

**Figure 6:**
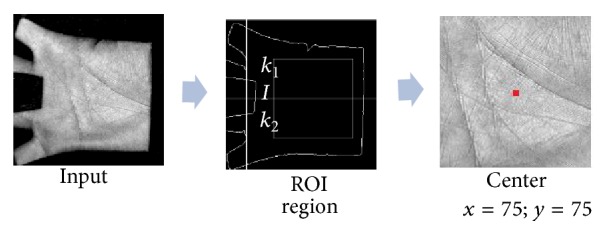
Localizing the center of ROI region of palm.

The center part of the palm print image is extracted as it contains prominent features such as wrinkles, ridges, and principal lines. The following are steps involved in ROI extraction:

1. Compute the centroid of a palm print and locate the point “I” between the middle finger and ring finger.
2. Take a 3 × 3 8-connectivity matrix by placing the pointer “P” (*P* is the center of mask) of the matrix at “I” and trace the corner points “*k*_1_” and “*k*_2_.”
3. Locate the midpoint “mid” between “*k*_1_” and “*k*_2_.”
4. Move from the point “mid” with the fixed number of pixels toward center of the palm and position the fixed sized square to crop the image and extract the subimage (ROI).
5. The center of ROI is the center of the fixed sized square to crop the image (Figure 6).

See [21] for a detailed description.

#### 3.2.4. Hand Shape

The segmentation of the hand silhouette is performed without requiring the extraction of any landmark points on the hand and this segmentation can be summarized as follows.

**Figure 7:**
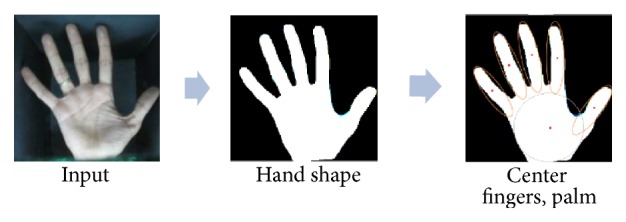
Localizing the center of fingers and palm.

After binarization, the first, the hand silhouette is segmented into six regions corresponding to the palm and the fingers. Segmentation is performed using an iterative process based on morphological filters [22]. The second is the geometric moment [19, 20] of each component of the hand that is considered for the calculation of five parameters; *X*_0_, *Y*_0_ denote the ellipse center; *θ* is the orientation; *α* and *β* are the minor and the major axes of the ellipse individually (Figure 4). Finally, the center of the best-fit ellipse of each component of the hand has been defined (Figure 7).

See [19, 20, 22] for a detailed description.

### 3.3. Feature Extraction with Zernike Moment

This module aims at extracting feature vectors or image-representing information. Features are extracted by ZM [15]. In our system, the extraction is performed on the derived images in parallel structure. That enables more characteristics of biometric images to be obtained.

#### 3.3.1. Zernike Moment

For a 2D image *f*(*x*, *y*), the image is changed from Cartesian coordinate into polar coordinate *f*(*r*, *θ*), where *r* and *θ* are radius and azimuth, respectively. The transformation of the images is done by the following formulae:

(8) r=x2+y2,

(9) θ=arctan⁡yx.

The image is specified on the unit circle with *r* ≤ 1 and enlarged by the basic functions *V*_*nm*_ = (*r*, *θ*).

Zernike Moment with order *n* and repetition *m* is defined as

(10) $$\begin{array}{c} M_{nm}=\frac{n+1}{\pi }\overset{2\pi }{\underset{0}{\int }}\overset{1}{\underset{0}{\int }}{\left[\;V_{nm}\left(\;r,\theta \;\right)\;\right]}^{*}f\left(\;r,\theta \;\right)r\;dr\;d\theta , \end{array}$$

where *∗* denotes complex conjugate, *n* = 0,1, 2,…, *∞*, and *m* is an integer subject to the constraint that *n* − |*m*| is nonnegative and even. *V*_*nm*_(*r*, *θ*), Zernike polynomial, is defined over the unit disk as follows:

(11) $$\begin{array}{c} V_{nm}\left(\;r,\theta \;\right)=R_{nm}\left(\;r\;\right)e^{im\theta } \end{array}$$

with the radial polynomial *R*_*nm*_(*r*) defined as

(12) Rnmr=∑s=0n−m/2−1sn−s!rn−2ss!n+m/2−s!n−m/2−s!.

The kernels of ZMs are a set of orthogonal Zernike polynomials so that any images can be represented by complex ZMs. Given all ZMs of an image, the image can be reconstructed as follows:

(13) $$\begin{array}{c} f\left(\;r,\theta \;\right)=\underset{n}{\sum }\underset{\left(\text{All }m\text{'}s\right)}{\sum }M_{nm}V_{nm}\left(\;r,\theta \;\right). \end{array}$$

The advantages of Zernike moments are translation, rotation, and scaling invariant. The invariant properties of Zernike moments are utilized as pattern sensitive features in recognition applications [27]. A short discussion about their invariant properties should be considered.

(1) Translation invariance can be obtained by converting the original image *f*(*x*, *y*) into the absolute pixel coordinates as follows $f(x+\overset{-}{x},y+\overset{-}{y})$, where $\overset{-}{x}=m_{10}/m_{00}$ and $\overset{-}{y}=m_{01}/m_{00}$ are the centroid coordinates of the original image (with m denoting the geometrical moment).

(2) Scaling invariance can be achieved by normalizing the Zernike Moment with respect to the geometrical moment *m*_00_ of the image. The improved Zernike moments are derived from the following equation: *Z*_*nm*_′ = *Z*_*nm*_/*m*_00_ with *Z*_*nm*_ are the Zernike moments of (10).

(3) Rotation invariance can be considered when *f*(*x*, *y*) is rotated by an angle *α*; we have the Zernike Moment *Z*_*nm*_ of the rotated image defined as

(14) $$\begin{array}{c} Z_{nm}^{\prime }=Z_{nm}e\left(\;-jm\alpha \;\right). \end{array}$$

In this way, the magnitudes of ZMs can be used as features of rotational invariances of an image.

#### 3.3.2. Feature Extraction

**Figure 8:**
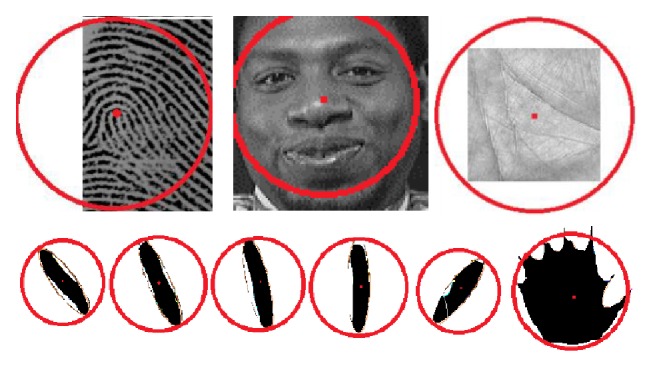
Example of ZM used for biometric images feature extraction.

In this phase, the center of the unit circle (the basis functions of ZM) in biometric images is determined. The center of the unit circle of ZM is set to coincide with the reference point in a fingerprint image, with the center of the best-fit ellipse in a face image (best-fit ellipse is an ellipse that encloses the facial region in a face image with frontal view), with the center of the circumscribed circle of square region in a palm table which is called region of interest (ROI), with the center of the best-ellipse-fitting of a palm and each finger (Figure 8).

Zernike Moment has shown in literature its ability to perform better than other moments (e.g., Tchebichef moment [30], Krawtchouk moment [31]). In fact, the increase in the orders of ZM will lead to a reduction in the quality of the reconstructed image due to the numerical changeability of ZM. Thus, in our work, the first 10 orders of ZM with 36 feature vector elements were chosen for a better performance of ZM.

### 3.4. Proposed Matching and Fusion

The sets of feature vectors obtained following image feature extraction are supplied to the matching modules, where they are matched with templates stored in the database. The Euclidean distance metric is applied to calculate similarity between the two feature vectors to generate matching scores.

In this work, we propose a supervised fusion where the classifiers (genuine or impostor) are trained using the match score densities and the parameters of the finite Gaussian mixture model that are used for modelling the genuine and impostor score densities of the training data.

According to the Neyman–Pearson theorem, the optimal test for deciding a score vector** x** to the class genuine or impostor is the likelihood ratio test given by

(15) $$\begin{array}{c} L\left(\;x\;\right)=\frac{f_{\text{gen}}\left(x\right)}{f_{\text{imp}}\left(x\right)}, \end{array}$$

where *f*_gen_(**x**) and *f*_imp_(**x**) are the estimated densities from the training data of genuine and impostor match scores, respectively. In this paper, the GMM automatically estimates the number of components and the component parameters using the Expectation-maximization (EM) algorithm [14] and the minimum message length criterion. The probability distribution for a *d*-dimensional object **x** is given by

(16) Nx=2π−d/2Σ−1/2exp⁡−12x−μTΣ−1x−μ,

where **x** is the match score vector, ***μ*** is the mean vector, and Σ is the covariance matrix of the training set. Assuming that both the genuine class and the impostor class have a mixture of Gaussian distributions, as expressed by

(17) fgenx=∑i=1Mgencgen,iNgen,ixfimpx=∑i=1Mimpcimp,iNimp,ix,

where *M*_gen_ (*M*_imp_) is the number of mixture components of the genuine (impostor) score and *c*_gen,*i*_  (*c*_imp,*i*_) is the weight assigned to the *i*th mixture component, ∑_*i*=1_^*M*_gen_^*c*_gen,*i*_ = ∑_*i*=1_^*M*_imp_^*c*_imp,*i*_ = 1.

## 4. Experimental Results and Discussion

### 4.1. Experimental Results

**Figure 9:**
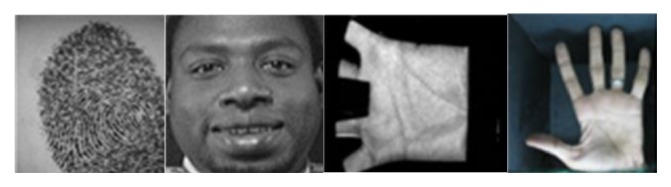
Some samples from the dataset used in this work.

Experiments have been conducted on several datasets. Brief information about the four used databases (Figure 9) is presented as follows:

1. FVC2004 fingerprint database [23]: FVC2004 DB4 includes 800 fingerprints of 100 fingers (8 images of each finger). Size of each fingerprint image is 288 × 384 pixels, and its resolution is 500 dpi.
2. ORL face database [24]: ORL is comprised of 400 images of 40 people with various facial expressions and facial details. All images were taken on dark background with a size of 92 × 112 pixels.
3. PolyU palm print database [25]: PolyU contains 7752 grayscale images corresponding to 386 different palms. Around 20 images per palm have been collected in two sessions. Size of each image is 384 × 284 pixels.
4. IIT Delhi hand shape database [26]: IIT Delhi has collected left and right hand images from 235 subjects. Each subject contributed at least 5 hand images from each of the hands. Size of each image is 800 × 600 pixels. From this dataset several biometric characteristics are segmented (palm, fingers, and hand shape). The palm and fingers are segmented using morphological operators proposed in [19, 20, 22].

In Table 1, we report the number of mixture found for the genuine data and for the impostor data in the four datasets used in this work.

In our experiment, the training set used for density estimation was formed with half of the genuine and half of the impostor match scores chosen randomly, and this division was repeated 10 times. As the achieved experimental results, the receiver operating characteristic (ROC) curves match the mean of genuine accept rate (GAR) values in all 10 tests conducted at different FAR values, and our proposed approach led to average verification accuracies in GAR.

**Figure 10:**
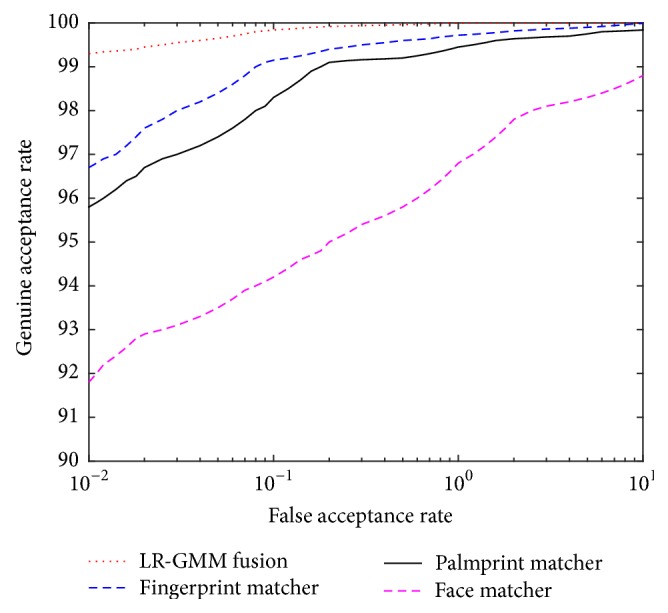
The ROC curves of the LR-GMM fusion and individual matchers.

The ROC curves of the LR-GMM fusion rule with four matchers and individual matchers in the FVC2004, the PolyU, and the ORL database are presented in Figure 10.

The performance of the LR-GMM fusion rule is significantly improved in comparison to the best individual modality from the four databases. LR-GMM fusion rule also brings about an increase in GAR with FAR of 0.01% (Table 2). Noticeably, the average verification accuracies presented in Table 2 show that the efficiency of the proposed method remained constant in 10 cross-validation trials and that multibiometric fusion of difference traits (fingerprint scores and palm print scores, fingerprint scores and face scores, and palm print scores and face scores) in the FVC2004, the PolyU, and the ORL databases considerably improved GAR compared to other multibiometric fusions (two fingerprint scores' fusion, two palm print scores' fusion, two face scores' fusion and hand scores' fusion).

**Figure 11:**
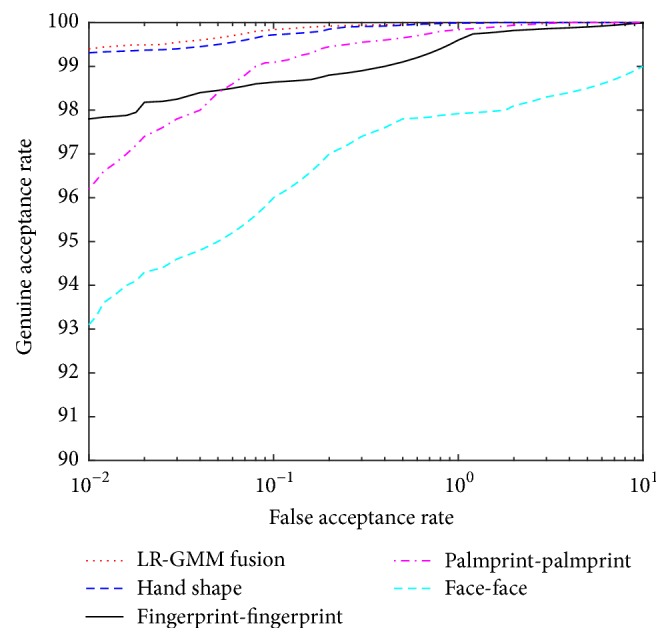
The ROC of LR-GMM on each database and on four databases.

The ROCs curves of LR-GMM fusion rule on four databases and LR-GMM fusion rule on each database (two fingerprint scores' fusion, two palm print scores' fusion, two face scores' fusion and hand scores' fusion) are presented in Figure 11.

**Figure 12:**
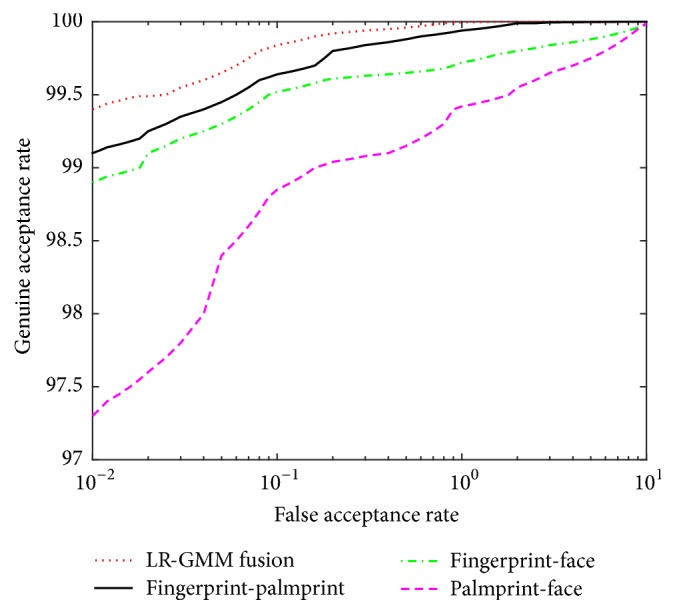
The ROC of LR-GMM on four databases and LR-GMM of difference traits.

The ROCs curves of LR-GMM fusion rule on four databases and LR-GMM fusion rule of difference traits (fingerprint scores and palm print scores, fingerprint scores and face scores, palm print scores and face scores) in the FVC2004, the PolyU, and the ORL database are shown in Figure 12.

According to our achieved experimental results, LR-GMM fusion can improve the GAR compared to the best individual modality. In particular, at the FAR of 0.01%, the mean GAR of LR-GMM fusion rules is 99.4% while the GAR values of the face, fingerprint, palm print, and hand shape modality are successively 93.2%, 97.8%, 96.3%, and 99.32%.

**Figure 13:**
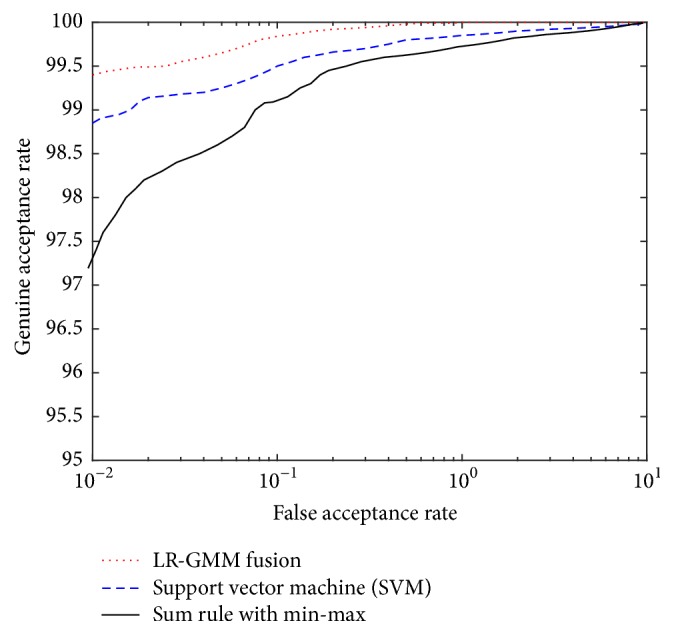
The ROC of LR-GMM, SVM, and Sum rule.

The performance of LR-GMM fusion rule was also compared with its performance using the support vector machine (SVM) classifier-based fusion, a classifier-based score fusion technique, and the sum of scores fusion method, a transformation-based score fusion technique. To enhance performance, the radial basis function (RBF) was chosen as the kernel function for SVM classifier. To use the sum of scores technique, the min-max normalization method [8] was used. We noted that the sum rule with min-max worked efficiently in our experiments on the chosen datasets. The ROC curves of the LR-GMM fusion rule, SVM classifier, and the sum rule with min-max on the multimodals of FVC2004, ORL, PolyU, and IIT Delhi are shown in Figure 13.

The proposed system was also compared with the other recognition systems, particularly face recognition system [27], fingerprint recognition system [28], palm print recognition system [29], and hand shape recognition system [22] using Zernike Moment and similar databases. The comparative results in Table 3 prove that the average verification accuracies at 0.01% FAR of our system can perform better than other recognition systems in terms of recognition rate.

### 4.2. Discussion

From the experimental results, some significant features of the proposed system using ZM-LR-GMM can be seen as below.

1. Determining the center of the biometric images will extract more features and increase the accuracy of the personal identification.
2. ZM is invariant to rotation, scale, and translation. Also, the feature extraction using Zernike Moment can provide feature sets with similar coefficients for easy computation.
3. The fusion rule using LR-GMM achieved high verification rate as well as easy implementation.
4. Our proposed method can work well on more databases.

Typically, there is a tradeoff between the additional cost and the improvement in performance of a multibiometric system. The cost could be the number of sensors deployed, the time required for acquisition and processing, performance gain (reduction in FAR/FRR), storage and computational requirements, and perceived convenience to the user.

## 5. Conclusion

In this paper, the authors have presented a novel feature extraction approach for the fusion of match scores in a multibiometrics system based on the likelihood ratio test and the finite Gaussian mixture model, in which biometric images are extracted by Zernike Moment to obtain comparable feature vectors. The proposed ZM-LR-GMM approach was tested on the publicly available databases such as FVC2004, ORL, PolyU, and IIT Delhi. It can be noted from the experiment that the fusion of comparable feature vectors contains more information about biometric images and thus can improve the verification rate. Practically, the highest verification rates GAR of 99.4% and FAR of 0.01% are achieved; this represents the outstanding performance of this proposed system. With its advantages, the proposed ZM-LR-GMM system can minimize lack of information and increase verification rate.

## Figures and Tables

**Table 1:** The number of mixtures for the genuine data and the impostor data.

	FVC2004-DB4 Fingerprint	ORL Face	PolyU Palm print	IITK hand shape	
				Palm	Fingers
Genuine	6	8	6	4	4
Impostor	6	8	6	4	4

**Table 2:** Performance achieved.

Database		Mean GAR at 0.01% FAR		
		Single matcher	LR-GMM	
			The same traits	Difference traits
	Multimodal		99.4%	99.4%
IIT-Delhi	Hand shape		99.32%	
FVC2004-DB4	Fingerprint	96.7%	97.8%	99.1% (fingerprint-palm print)
PolyU	Palm print	95.8%	96.3%	98.9% (fingerprint-face)
ORL	Face	91.8%	93.2%	97.3% (palm print-face)

**Table 3:** Accuracy rate achieved by different algorithms.

	Ours	Exist
Face	93.2%	92.8% [27]
Fingerprint	97.8%	92.89% [28]
Palm print	96.3%	91.25% [29]
Hand shape	99.32%	99.3% [22]
Proposed LR-GMM fusion	99.4%	
